# Smart Nanoparticles Are Not Smart Enough (Yet): A Cell-Aware View of Cancer Nanomedicine

**DOI:** 10.3390/cells15060491

**Published:** 2026-03-10

**Authors:** Serena Marchiò

**Affiliations:** 1 Department of Oncology, University of Turin, 10060 Candiolo, Italy; serena.marchio@unito.it; Tel.: +39-01199333239; 2 Candiolo Cancer Institute, Fondazione del Piemonte per l’Oncologia—Istituto di Ricovero e Cura a Carattere Scientifico (FPO-IRCCS), 10060 Candiolo, Italy

**Keywords:** cancer nanomedicine, smart nanoparticles, cellular stress responses, cell-state plasticity, functional profiling, omics-based analysis, tumor microenvironment

## Abstract

“Smart” nanoparticles are often presented as the vanguard of precision cancer therapy, defined by engineered abilities to sense predefined stimuli, enhance targeting, and control therapeutic release. Yet this notion of smartness remains largely material-centric and only partially reflects how nanomedicines behave in vivo. Cells exposed to nanoparticles are not passive recipients of engineered functions; they actively interpret these perturbations through integrated stress-response, metabolic, transcriptional, and innate immune programs. These cell-state trajectories can determine efficacy, tolerance, resistance, or toxicity, and can do so independently of uptake, biodistribution, or triggerable release efficiency. Accordingly, evaluation strategies that prioritize delivery metrics and limited a priori molecular markers may misestimate functional performance and durability. This Perspective proposes a cell-aware reframing in which smartness is defined by biological controllability: the capacity of a nanoparticle system to elicit predictable, mechanistically interpretable, and therapeutically favorable cell-state trajectories across relevant malignant and non-malignant compartments. A practical path forward is to integrate time-resolved functional profiling into benchmarking using compact response signatures that report stress buffering, immune activation or suppression, and the emergence of tolerant states. A practical path forward is to integrate time-resolved functional profiling into benchmarking using compact response signatures that report stress buffering, immune activation or suppression, and the emergence of tolerant states. Here, biological controllability refers to the ability of a nanoparticle system to reproducibly steer integrated cellular stress, metabolic, and immune programs toward predefined therapeutic endpoints while minimizing adaptive escape across heterogeneous compartments.

## 1. Introduction

Cancer nanomedicine has progressed from conventional drug carriers to increasingly sophisticated platforms engineered for conditional behavior in complex biological environments. Contemporary “smart” nanoparticles integrate features such as ligand-mediated targeting, microenvironment-responsive release, biomimetic cloaking, and theranostic functions, aiming to improve tumor specificity and therapeutic index [1]. These advances have been driven largely by rational materials design, with smartness typically framed as cue-responsive, programmed behavior.

However, tumors are not passive landscapes for engineered materials to navigate. Cancer cells, stromal cells, and immune cells actively perceive and respond to nanoparticle exposure [2,3] through interconnected signaling networks that govern stress adaptation, metabolic homeostasis, transcriptional reprogramming, and phenotypic plasticity [4,5,6,7,8]. Such cellular programs can shape therapeutic outcomes at least as strongly as delivery efficiency itself. In this context, a nanoparticle that performs as designed (e.g., accumulates in tumors, internalizes efficiently, releases cargo under a trigger) may still fail to produce durable benefit if the induced cellular response favors tolerance, adaptive resistance, or compensatory survival states.

**Figure 1:**
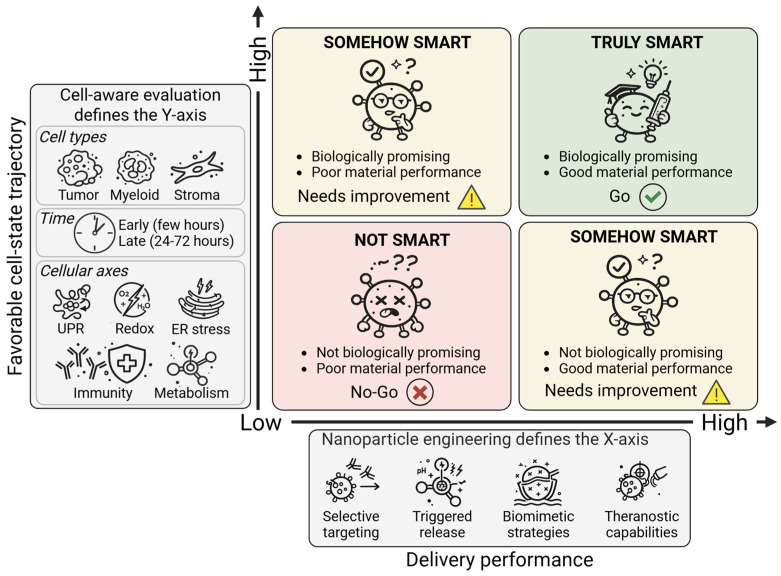
A cell-aware decision framework for defining smartness in cancer nanomedicine. Nanoparticle performance can be evaluated along two orthogonal dimensions: delivery performance, defined by nanoparticle engineering features (*X*-axis), and the favorability of induced cell-state trajectories, defined by cell-aware evaluation (*Y*-axis). When only one of these two features is consolidated, efficient and durable therapeutic benefit is not guaranteed (“SOMEHOW SMART”). True smartness emerges when robust nanoparticle engineering is coupled to predictable, mechanistically interpretable, and therapeutically favorable cellular responses across malignant and non-malignant compartments (“TRULY SMART”). UPR, unfolded protein response; ER, endoplasmic reticulum.

This mismatch between material-centric definitions of smartness and cell-state determinants of outcome may contribute to inconsistent performance across models [9] and to persistent gaps between preclinical promise and clinical translation [10]. Standard evaluation pipelines often emphasize accumulation, uptake, and selected pathway markers, providing an incomplete view of the dynamic and heterogeneous cell-state trajectories elicited by nanoparticle exposure [11,12,13]. A reframing is therefore warranted: rather than asking only whether a nanoparticle is “smart” by design, it becomes essential to ask whether it is smart in biological terms, namely, whether it induces controllable, interpretable, and therapeutically favorable cell-state trajectories across relevant cell types and contexts (Figure 1). This view predicts that formulations optimized by delivery metrics alone will often diverge in durability once their cell-state trajectories are measured across relevant compartments. Recent perspectives have already emphasized the limitations of delivery-centric paradigms and called for broader systems-level analyses of nano–bio interactions. These efforts have expanded analytical resolution but have generally framed smartness as an engineering attribute evaluated with deeper measurement. The present Perspective differs in proposing a definitional shift: smartness should be judged by the reproducible steering of cell-state trajectories across relevant compartments. In this view, functional profiling is not an added layer of characterization, but the means to determine whether engineered responsiveness translates into biologically controllable outcomes.

This Perspective revisits the concept of smart nanoparticles through cell biology, proposing cellular interpretation as a core dimension of nanoparticle function, with implications for how smart systems are defined, evaluated, and optimized for cancer therapy.

## 2. What Do We Mean by “Smart” Nanoparticles?

The term “smart nanoparticles” is broadly used to describe nanoplatforms engineered to perform context-dependent functions in response to predefined cues. In cancer nanomedicine, smartness most commonly refers to one or more of the following design attributes: (i) selective accumulation or binding through passive or ligand-mediated targeting; (ii) conditional activation or release triggered by endogenous cues (pH, redox state, enzymatic activity, hypoxia) or external energy inputs (heat/light, magnetic fields, ultrasound); (iii) biomimetic strategies intended to improve circulation, immune evasion, or tissue penetration; and (iv) theranostic capabilities that combine therapy with imaging or sensing [14,15,16]. In practice, these features are typically validated using delivery-centric benchmarks such as biodistribution, uptake, intracellular trafficking, and triggerable release profiles [17].

This prevailing definition is material-centric: smartness is inferred from nanoparticle behavior under controlled conditions. Yet in living systems, the functional meaning of a “smart” behavior depends on how cells and tissues interpret it [4]. A stimulus-responsive release mechanism may be technically elegant while producing limited or variable biological impact if exposed cells engage buffering stress responses, shift metabolic programs, or enter tolerant phenotypic states. Conversely, nanoparticles lacking overt “smart” triggers may still exert strong biological effects by perturbing membrane dynamics, endo-lysosomal trafficking, redox balance, or innate immune sensing pathways.

From a cell biology perspective, smartness can be viewed as an emergent property of a bidirectional interaction: engineered nanoparticle behavior on one side, and cellular interpretation on the other. Cells integrate physical and chemical perturbations into coordinated programs that shape proliferation, death, immune signaling, and adaptive plasticity; in cancer, heterogeneity and stress adaptation can make these programs decisive for therapeutic outcome. Accordingly, a more complete definition of smart nanoparticles should extend beyond engineered responsiveness to include the ability to elicit predictable, mechanistically interpretable, and therapeutically favorable cell-state trajectories.

This reframing does not diminish the importance of rational nanoparticle engineering; rather, it clarifies the endpoint that engineering should serve. If smartness is intended to enable precision therapy, then the relevant unit of precision is not only tumor localization but also the cellular programs engaged upon exposure. Treating cellular interpretation as a core functional dimension provides a basis to reassess how smart nanomedicines are benchmarked and why design sophistication does not always translate into consistent biological or clinical benefit [11]. The conceptual contribution here is not to further catalog nano–bio interactions, but to reposition controllable cell-state trajectory steering as the defining criterion of smartness. Heterogeneity is treated not as noise to be averaged, but as a decision-relevant signal revealing structured responder and tolerant states. Smartness thus becomes measurable by the reproducibility and favorability of induced biological paths, rather than by stimulus responsiveness alone.

## 3. Cells Are Smarter than the Nanoparticles

Cells are equipped with robust sensing and control systems that detect perturbations and reconfigure functional states to preserve viability, reshape behavior, or initiate immune signaling. Nanoparticle exposure (whether intended as a delivery event or not) constitutes a multi-parameter perturbation involving particle physicochemical properties (size, charge, surface chemistry, mechanics), corona-mediated identity, membrane interactions, trafficking routes, and endo-lysosomal processing [18,19,20]. These inputs are integrated into coordinated response programs that can dominate downstream therapeutic outcomes. Importantly, such programs can be triggered not only by the payload but also by the nanoparticle carrier and its intracellular processing [4].

Across malignant and non-malignant compartments, nanoparticle exposure can engage conserved cellular axes including oxidative stress and redox buffering, proteostasis/endoplasmic reticulum stress (unfolded protein response), DNA damage signaling, lysosomal remodeling and autophagy dependence, innate immune/inflammatory sensing, and metabolic adaptation [5,21,22]. The direction and magnitude of these responses are context-dependent: they vary with baseline cellular state, oncogenic background, nutrient availability, oxygen tension, and prior stress history. As a consequence, the same nanoparticle formulation can produce qualitatively different outcomes across cancer cell subtypes, fibroblasts, endothelial cells, macrophages, dendritic cells, or T cells, even when uptake appears comparable.

This adaptive capacity is particularly relevant in cancer, where phenotypic plasticity and non-genetic heterogeneity enable rapid state transitions under therapeutic pressure. Nanoparticle-delivered cytotoxics or targeted agents may transiently reduce viability while simultaneously selecting for tolerant subpopulations characterized by altered metabolism, stress tolerance, quiescence-like programs, or enhanced drug efflux [6]. Similarly, immunomodulatory payloads may trigger immune activation in one cellular context but induce compensatory immunosuppressive programs in another, depending on baseline inflammatory tone and antigen presentation states. In both cases, adaptive capacity is expressed as the ability to absorb perturbations and redirect trajectories—sometimes toward the intended effect, often toward accommodation or escape.

Particle composition and intracellular processing can perturb ionic balance, redox homeostasis, membrane integrity, and trafficking dynamics, generating biological effects that are only partially captured by classical delivery metrics. This is especially relevant for repeated dosing, where chronic low-level stress signaling or innate immune activation can reshape tissue states over time, altering subsequent nanoparticle responses and therapeutic windows [7,23,24].

## 4. When Smart Design Meets Delivery-Dominant Evaluation

Despite increasingly sophisticated nanoparticle engineering, the biological evaluation of smart nanomedicines often remains narrow relative to the complexity of the cell-state changes they induce. Standard pipelines prioritize delivery-centric endpoints (circulation time, tumor accumulation, uptake efficiency, intracellular localization, and stimulus-triggered release) because these metrics are measurable, comparable, and tightly linked to design parameters [11,25,26]. Yet these readouts describe what the nanoparticle does, not what exposed cells become. In cancer, where adaptive state transitions frequently determine treatment failure, this distinction is causal rather than semantic.

Biological efficacy is commonly inferred from short-term viability assays, tumor growth delay, and a limited set of molecular markers selected a priori (e.g., apoptosis indicators, cytokines, pathway phosphorylation). Such measurements can confirm that a payload is active, but they often miss system-level adaptation. Stress buffering, metabolic rewiring, shifts in differentiation state, and the emergence of slow-cycling tolerant subpopulations can unfold over time and remain invisible at early endpoints. A formulation can therefore appear “smart” by delivery and acute efficacy criteria while inducing cell-state trajectories that erode durability.

Heterogeneity magnifies this problem. Identical nanoparticles can elicit divergent responses across cell types within the tumor microenvironment and across subpopulations within the same tumor. Delivery metrics averaged across tissues or bulk tumor samples can mask opposing programs—for example, tumor-cell tolerance emerging alongside macrophage activation, or high uptake coinciding with endo-lysosomal sequestration and limited cytosolic bioavailability [27,28,29]. In such cases, the apparent alignment between design intent and biological effect becomes model-dependent and difficult to generalize, limiting translational predictability.

A further blind spot lies in attributing outcomes primarily to the payload while treating the nanoparticle as inert. Particle properties and intracellular processing can themselves activate innate sensing, perturb redox homeostasis, remodel lysosomes, or alter trafficking dynamics, all effects that may synergize with, antagonize, or outlast payload action [8]. Without systematic interrogation of these cell-intrinsic responses, mechanistic interpretations can become overly linear (“trigger → release → kill”) and may incompletely capture adaptation, tolerance, or context-specific toxicity.

Divergence between delivery efficiency and biological response is increasingly evident. Lipid nanoparticles engineered for robust endosomal escape can achieve efficient cytosolic delivery yet simultaneously activate innate sensing or stress pathways that constrain repeat dosing or reshape tissue states. Similarly, clinical formulations such as liposomal doxorubicin improved pharmacokinetics and reduced systemic toxicity relative to free drug, but produced variable survival benefits across tumor types [30]. In both cases, engineering success at the level of biodistribution or trafficking did not uniformly translate into durable state reprogramming.

Taken together, these limitations suggest that smart nanoparticle design is frequently paired with evaluation frameworks that are incompletely aligned with biological complexity at the level of cell biology. If the goal is precision and durability, assessment must move beyond whether the material behaves as intended and ask whether cell-state trajectories follow favorable and reproducible paths across relevant compartments (Figure 1). In practical terms, the missing layer is time-resolved, multi-compartment functional profiling—an addition that can complement delivery benchmarks and guide biologically informed optimization.

## 5. Toward Cell-Aware Smart Nanomedicine

If cellular interpretation is a primary determinant of nanoparticle performance, then smart nanomedicine requires evaluation frameworks that treat cellular state as a first-class endpoint. A cell-aware approach does not replace rational materials design; it refocuses the target of optimization. Instead of engineering particles only to sense microenvironmental cues or improve accumulation, the objective becomes to elicit predictable and therapeutically favorable cell-state trajectories across relevant malignant and non-malignant cell types [31].

A practical route forward is to integrate functional response profiling into nanoparticle benchmarking [4,32]. Rather than relying predominantly on uptake, release, and selected pathway markers, assessment can incorporate response “signatures” that capture integrated stress, metabolic, inflammatory, and differentiation programs [33,34]. Transcriptomic, proteomic, and metabolic readouts paired with targeted phenotypic assays (growth dynamics, death modalities, senescence-like programs, autophagy/lysosomal remodeling, innate immune activation) can map how cells reconfigure over time in response to a nanoparticle formulation [4,33,34]. The goal is not exhaustive cataloging, but identification of interpretable response axes that associate with durable efficacy or with adaptation and toxicity [32].

Time and context should be treated as design variables. Early responses may reflect acute stress, whereas later time points reveal whether cells recover, adapt, or commit to irreversible fates. Profiles should also extend beyond a single cancer cell line to include cell types that shape therapeutic outcome, such as macrophages, dendritic cells, endothelial cells, fibroblasts, and T cells [31,35]. Even limited panels can reveal whether a formulation promotes immune activation versus immunosuppression, vascular disruption versus repair, or tumor cell killing versus tolerance programs. This is especially relevant for biomimetic and immunomodulatory nanoparticles, where the same surface features may produce opposing consequences across cellular compartments [31]. Evidence from drug tolerance studies indicates that the magnitude, duration, and coordination of stress-response programs can be associated with divergent long-term outcomes. Transient activation of pathways such as unfolded protein response or interferon signaling may accompany irreversible commitment, whereas buffered or reversible activation can permit entry into tolerant persister states [36]. These observations suggest that trajectory shape, and not merely pathway presence, may carry predictive value, reinforcing the need for time-resolved profiling [37].

Cell-aware profiling also reframes heterogeneity as actionable information. Variability across subpopulations can reveal structured “responder” and “tolerant” states, enabling stratification of models and identification of combinations that constrain adaptation. For example, if a nanoparticle consistently induces oxidative stress buffering or autophagy-dependent survival, rational co-interventions can be tested to block these escape routes. Conversely, if efficacy correlates with specific cellular programs (e.g., immunogenic stress, antigen presentation, interferon signaling), design choices can be evaluated by their ability to engage these trajectories rather than by delivery metrics alone [35].

Finally, this framework supports an iterative feedback loop between engineering and biology [38]. Response signatures can be compared across formulations to determine which particle properties drive favorable versus unfavorable cellular programs, guiding design refinement with mechanistic direction. In this sense, smartness becomes measurable as biological controllability: the capacity of a nanoparticle system to steer cell-state trajectories toward outcomes that are reproducible across contexts and resilient to adaptive escape.

## 6. Implications for Translation and Precision Oncology

A cell-aware definition of smart nanomedicine has direct implications for why clinical translation remains inconsistent and how it may be improved without relying solely on incremental materials innovation [11,12]. Many translational failures are interpreted as delivery problems, namely, insufficient tumor accumulation, heterogeneous penetration, or unfavorable pharmacokinetics. While these barriers are real, they do not fully explain why nanoparticles that meet design benchmarks can produce variable efficacy, unexpected toxicity, or limited durability across patients. A complementary explanation is that cellular interpretation differs across tumors and tissues, generating distinct cell-state trajectories even under comparable exposure.

From a translational standpoint, functional cellular responses can serve as bridging biomarkers between preclinical models and clinical behavior [31]. A practical implementation could be a compact response panel capturing stress buffering, innate immune activation, and tolerant-state markers measured longitudinally in tumor and myeloid compartments. If a nanoparticle formulation reproducibly induces stress-tolerant programs, immunosuppressive signaling, or metabolic compensation, these signatures may predict limited benefit or rapid relapse, regardless of delivery efficiency [6]. Conversely, formulations that elicit favorable trajectories, such as sustained apoptotic commitment, immunogenic stress programs, or coordinated immune activation across relevant compartments, may be more likely to achieve durable responses. This framing encourages the development of biologically grounded go/no-go criteria that extend beyond biodistribution and acute tumor shrinkage.

Cell-aware profiling also clarifies the logic of personalization. Precision nanomedicine is often conceived as matching a particle to a tumor based on receptor expression or microenvironmental cues [39]. Yet receptor presence does not guarantee a favorable cellular response, and tumors sharing a receptor can differ markedly in adaptive capacity. Personalization may therefore require stratifying patients by baseline cellular states and response potential (stress sensitivity, metabolic dependencies, immune contexture, and plasticity) rather than by target expression alone [6,31]. Even modest state-based stratification could improve trial design by enriching for tumors in which a given nanoparticle is more likely to drive favorable trajectories. Cell-state profiling should complement, not replace, genomic biomarkers. Genomic alterations define potential vulnerabilities, whereas dynamic response profiling captures adaptive capacity and stress-buffering potential. Tumors sharing a targetable mutation may nonetheless diverge in trajectory upon nanoparticle exposure. Integrating static genomic context with dynamic state assessment may therefore refine patient stratification by identifying tumors that are both target-positive and state-permissive for durable reprogramming [40].

Combination strategies can also be reframed. Nanoparticles are frequently proposed as delivery vehicles for combination therapy; however, combinations are often selected based on pharmacologic synergy or pathway rationale without explicitly considering nanoparticle-induced adaptation [41]. If profiling reveals that a formulation induces specific escape programs (e.g., autophagy dependence, antioxidant buffering, innate immune desensitization), combinations can be chosen to constrain these trajectories, turning adaptation mapping into a rational guide for durable control [6,41].

Finally, a cell-aware framework may improve safety assessment. Off-target toxicity is typically framed as exposure of healthy tissues to payload. Yet toxicity can also arise from cell-state perturbations induced by the nanoparticle system itself, including chronic stress signaling, inflammatory activation, or immune dysregulation after repeated dosing [24]. Profiling responses in relevant non-malignant cell types can therefore identify liabilities earlier and support safer design iteration [11,24].

In sum, translation and precision in cancer nanomedicine may depend less on perfecting a universal “smart” platform and more on establishing predictable relationships between nanoparticle properties and cellular state outcomes [7,8]. Treating cellular interpretation as a translational bridge offers a path to more informative preclinical benchmarks, more rational patient stratification, and more durable therapeutic impact [11,12].

## 7. Operationalizing Cell-Aware Smartness: A Practical Roadmap

A cell-aware framework is only useful if it can be implemented without turning nanoparticle evaluation into an open-ended profiling exercise [32,42]. The goal is therefore not maximal measurement, but a minimal, scalable set of experiments that reveals whether a formulation induces favorable cell-state trajectories and avoids predictable adaptive escape programs. Several practical principles can help operationalize “cell-aware smartness” in routine preclinical workflows (Figure 1). Operationalizing this framework does not require exhaustive multi-omics for every candidate formulation. Compact transcript panels, high-content imaging, and modular stress-response assays can be integrated into existing pipelines with tiered depth proportional to translational intent. As occurred with genomic and immunologic biomarkers, standardization and scalability are likely to emerge progressively rather than simultaneously.

1. Define a minimal cellular panel that reflects therapeutic reality. At minimum, profiling should extend beyond a single cancer cell line to include non-malignant compartments that shape efficacy and toxicity [42,43]. A pragmatic starting panel for many indications includes: (i) tumor cells representing relevant genotypes or phenotypic states; (ii) macrophages or monocyte-derived cells as primary innate immune interpreters of nanoparticles; and (iii) an endothelial and/or fibroblast component as key determinants of vascular transport, extracellular matrix dynamics, and inflammatory remodeling. Even a small panel can expose cell-type-specific liabilities (e.g., immunosuppressive polarization, endothelial stress responses) that are invisible in tumor-only assays [43].
2. Treat time as a first-class variable. Cellular trajectories cannot be inferred from a single endpoint. A minimal time-resolved design can separate acute stress from durable adaptation, for example, by sampling an early window (hours) and a later window (24–72 h), with optional extension to repeated exposures when clinically relevant [42,44]. Early responses often reveal primary sensing and damage signals, whereas later time points reveal whether cells recover, adapt, enter tolerant states, or commit to irreversible fates. This distinction is central to durability.
3. Track a small set of response axes rather than isolated markers. Cell-aware readouts can be anchored around interpretable biological modules that are frequently engaged by nanoparticle exposure and are linked to efficacy, tolerance, or toxicity [4]. A minimal set of broadly applicable axes includes: (i) redox/oxidative stress buffering; (ii) proteostasis and ER stress (unfolded protein response); (iii) lysosomal remodeling and autophagy dependence; (iv) inflammatory and innate immune sensing (including interferon-related programs); and (v) metabolic rewiring (e.g., shifts in glycolysis/oxidative metabolism or lipid handling). These axes can be monitored using compact transcript panels, targeted protein readouts, and orthogonal phenotypic assays, with full omics reserved for deeper dives when needed [4,32].
4. Separate particle effects from payload effects. Many mechanistic interpretations implicitly attribute cellular responses to the therapeutic cargo, while the nanoparticle is treated as inert [8]. A minimal design should include controls that enable decomposition of responses into carrier-only, payload-only (when feasible), and carrier-plus-payload conditions. This separation becomes especially important under repeated dosing, where nanoparticle-driven innate sensing or stress adaptation can shape subsequent responses and alter therapeutic windows.
5. Use state trajectories to define go/no-go criteria. Delivery metrics remain necessary but should not be sufficient [42]. Practical decision points can be based on whether a formulation: (i) induces durable cytotoxic commitment versus reversible growth arrest; (ii) promotes tolerant/escape states (e.g., stress-tolerant quiescence-like programs) versus irreversible loss of fitness [6]; and (iii) drives immune-supportive trajectories (e.g., antigen presentation, interferon signaling) versus immunosuppressive polarization or desensitization [7,23]. The key is to predefine what constitutes a favorable trajectory for the intended mechanism and indication, then evaluate formulations against that biological target [42].
6. Leverage heterogeneity rather than average it out. Where possible, profiling should capture variability across subpopulations, as average responses can mask the emergence of tolerant states. Even limited single-cell or high-content approaches can reveal whether a formulation creates a resistant tail, shifts the distribution of states, or selects for specific adaptive programs [32,44]. In a cell-aware framework, heterogeneity is not noise—it is an early warning signal for durability.
7. Iterate engineering using biological feedback. The endpoint of profiling is design refinement [38]. Response signatures can identify which particle properties consistently associate with unfavorable programs (e.g., chronic stress buffering, immunosuppressive polarization) and which correlate with favorable trajectories. This enables an iterative loop in which engineering choices are guided by cellular interpretation rather than by delivery performance alone.

In combination, these steps provide a feasible route to embed cell-state readouts into nanoparticle development pipelines. Operationalizing cell-aware smartness in this manner reframes optimization from maximizing delivery features to achieving controllable biological outcomes, and this is an adjustment that may be essential to improve translational robustness and therapeutic durability in cancer nanomedicine.

## 8. Limitations

This framework carries practical challenges. Expanding evaluation toward dynamic state profiling increases experimental complexity and may introduce variability across models. Interpretation of high-dimensional data requires careful standardization and restraint to avoid overextension of mechanistic claims. Moreover, profiling depth should remain proportional to indication and translational stage. These considerations reflect a maturation step rather than a barrier, analogous to earlier transitions in genomics and immuno-oncology before standardization.

## 9. Concluding Remarks: Rethinking Smartness in Cancer Nanomedicine

“Smart” nanoparticles have become a defining aspiration in cancer nanomedicine, yet smartness is still most often assigned based on engineered features (targeting ligands, triggerable release, or stimulus responsiveness) rather than on the biological trajectories these systems induce in living cells. As argued throughout this Perspective, therapeutic outcomes frequently depend on cellular interpretation: integrated stress-response, metabolic, inflammatory, and immune programs that can amplify intended effects or generate tolerance, resistance, and toxicity. In this setting, design sophistication does not guarantee biological control.

A cell-aware definition of smartness reframes the field around a more demanding (but more actionable) criterion: the ability of nanoparticle systems to elicit predictable, mechanistically interpretable, and therapeutically favorable cell-state trajectories across relevant cell types and contexts. Implementing this shift requires expanding evaluation beyond delivery-centric metrics toward functional profiling that captures state dynamics over time, distinguishes particle- from payload-driven effects, and treats heterogeneity as an informative signal rather than a confounder.

Ultimately, the next leap in cancer nanomedicine may not come from making nanoparticles incrementally smarter by design, but from making their biological consequences more controllable and more predictable. Treating cellular interpretation as a core dimension of smartness offers a path to align engineered responsiveness with durable therapeutic impact and to move closer to truly precision-oriented nanotherapy.

## Data Availability

No new data were created or analyzed in this study.

## Abbreviations

The following abbreviations are used in this manuscript:

ER	Endoplasmic reticulum
UPR	Unfolded protein response
